# Iatrogenic Submandibular Duct Rupture Complicating Sialography: A Case Report

**DOI:** 10.5812/ircmj.7882

**Published:** 2014-09-05

**Authors:** Hadi Sharouny, Rahmat Bin Omar

**Affiliations:** 1 Department of Otorhinolaryngology Head and Neck Surgery, Faculty of Medicine, Shiraz University of Medical Sciences, Shiraz, IR Iran; 2 Department of Otorhinolaryngology Head and Neck Surgery, Faculty of Medicine, University of Malaya, Kuala Lumpur, Malaysia

**Keywords:** Salivary Gland Calculi, Sialography, Salivary Ducts, Rupture

## Abstract

**Introduction:**

Sialolithiasis is the most common disease of salivary glands. Sialography is particularly important for the assessment of the outflow tract and in diagnosing obstructive salivary gland lesions including calculi.

**Case Presentation:**

We report on a 38-year-old female with sialolithiasis whom had Wharton’s duct perforation, complicating the sialography. She was treated conservatively with a course of co-amoxiclav, oral prednisolone for three days and pain-killers. The patient was clinically well on follow-up reassessments at the end of the first week and three weeks post procedure.

**Conclusions:**

Perforation of salivary duct complicating the sialography is rare. Awareness of this potential complication and utilizing a good sialography technique need to be advocated amongst radiologists. Response to treatment by conservative management is preferred as illustrated in this case.

## 1. Introduction

Sialolithisis is the most common disease of the salivary glands. In a recent study, based on hospital admission figures in England, Escudier and McGurk, incidence has been estimated between 1 per 15,000 and 1 per 30,000 (1). Imaging of salivary glands for stones may be accomplished with a plain radiograph, conventional and digital sialography, ultrasound (US), computerized tomography scan (CT-scan) and magnetic resonance imaging (MRI) (2).

Amongst these, sialography is an important tool for the assessment and diagnosis of salivary gland obstructions including those caused by calculi. This study was performed by a radiologist who infused a contrast agent into the Wharton’s duct after dilation of its opening was performed. Contrast material is injected slowly with no significant force and then the radiographs were taken. The procedure is well tolerated by most patients and complications are uncommon. 

## 2. Case Presentation

A 38-year-old woman was referred to our outpatient ear, nose and throat (ENT) clinic with the right submandibular swelling for about a one-year duration. Besides a mild intermittent ache during meals, she had no other relevant symptoms. The patient did not have any trauma or chronic medical illnesses. Clinical examination revealed a mass of about 2.0 × 2.0 cm at the right submandibular region which was firm in consistency. No tenderness was elicited or warmth felt on palpation. Fine needle aspiration cytology (FNAC) of the mass was ordered and the result was suggestive of sialadenitis. Sialography was then scheduled for further investigation of the lesion.

**Figure 1.**
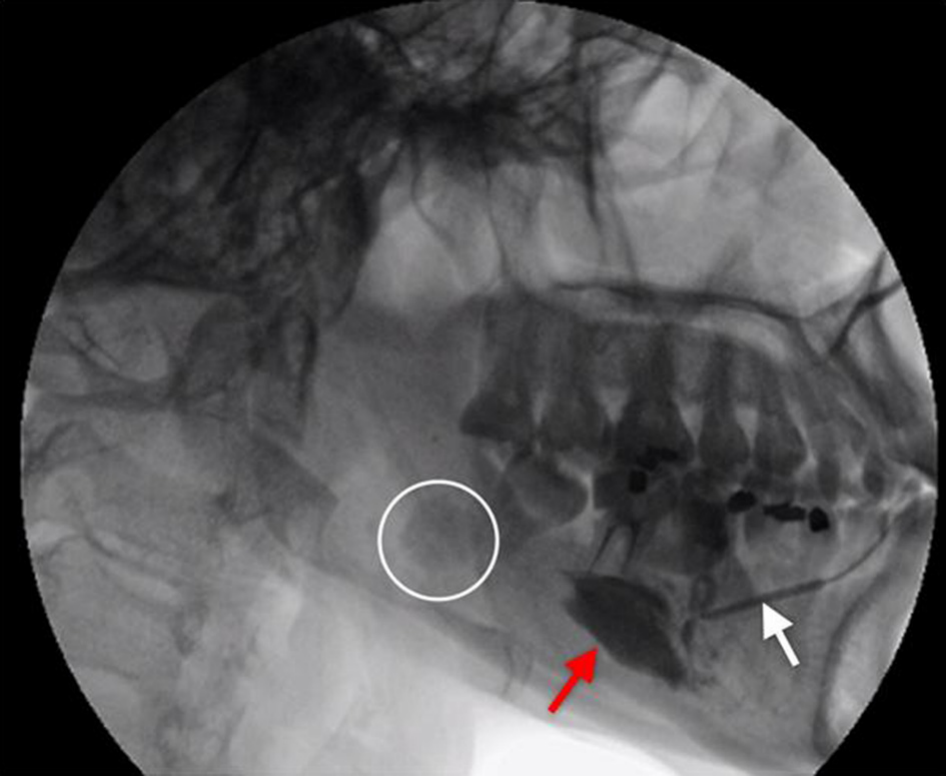
Extravasation of the contrast agent (red arrow) following submandibular duct rupture during sialography; an intra-glandular radiopaque calculus is seen (circle). White arrow indicates the submandibular duct.

During sialography, serial dilatation of right submandibular duct was performed, followed by insertion of size 16 F cannula. Furthermore, 3 mL of Ultravist (contrast agent) was injected and serial images were taken. No resistance was felt during infusion of the contrast material. The patient tolerated the procedure well and did not complain about having any pain. Opacification of the distal part of the submandibular duct was noted, followed by fusiform dilatation of the duct and extravasation of contrast (Figure 1). The patient was then immediately referred to an otolaryngologist.

**Figure 2.**
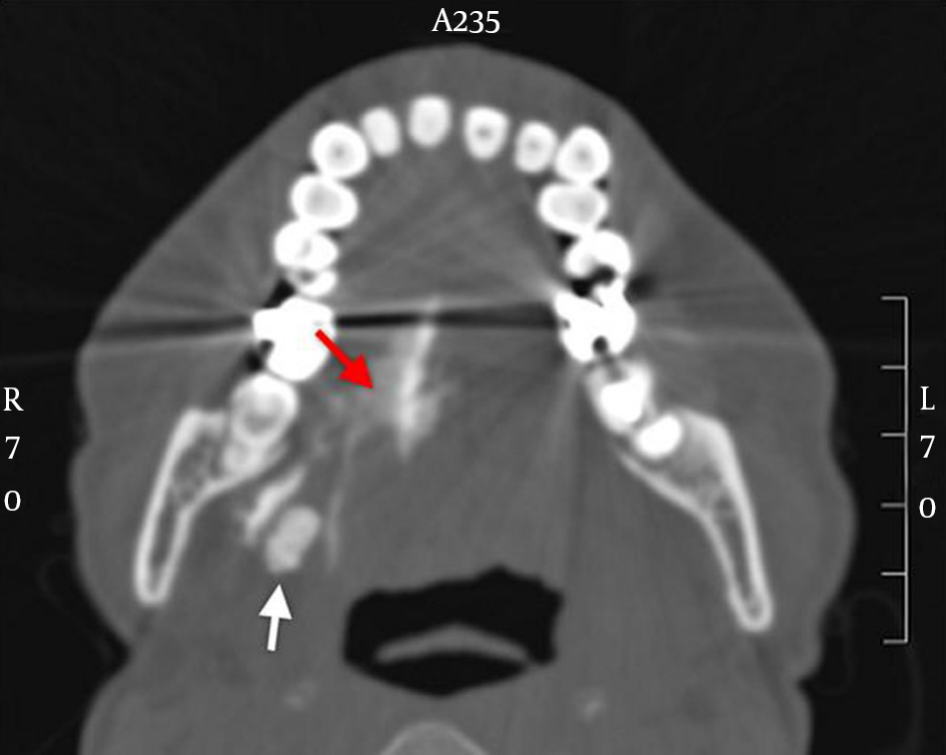
Figure shows a bone window axial cut view of the floor of the mouth CT-scan. A radiopaque submandibular stone (white arrow), and contrast extravasation into the surrounding soft tissues (red arrow) were well seen.

On bimanual examination, a stone could be felt in the floor of the mouth. A nodular mass of about 2.0 cm was palpated at the right submandibular region, which was non-tender. The CT scan of the submandibular gland was suggested by the otolaryngologist. The CT scan showed a radiopaque submandibular stone measuring 11.0 mm × 6.0 mm and right submandibular duct perforation as evidenced by contrast extravasation into soft tissue of the submandibular region (Figure 2). The patient was treated conservatively with amoxicillin/clavulanic acid (co-amoxiclav) for a week and prednisolone 60 mg for three days. The patient was well on the follow-up session a week later and no tenderness was found on palpation. Repeat lateral neck X-ray showed that the contrast material had disappeared. She was seen again at the ENT clinic, three weeks later and submandibular gland excision was scheduled.

## 3. Discussion

Sialolithiasis is the formation of calculi in the tree-like ductal system of the salivary glands. Its peak incidence is in middle aged adults (3) with women preponderance (2, 3). It accounts for more than 50% of the salivary gland diseases (4). Most of salivary calculi (more than 80%) occur in the Wharton’s duct (3, 4). The submandibular gland is more predisposed to sialolithiasis due to higher salivary alkaline content, has a greater concentration of calcium and phosphate, higher mucus content, its duct is longer with a kink at the rear part of the floor of the mouth, and also has antigravity flow of saliva (5).

The most common symptoms of calculi of salivary glands are recurrent pain and swelling of the affected gland during meals (2, 6). The patient may have a history of acute suppurative sialadenitis (2, 3). On physical examination, bimanual palpation will show a palpable stone in most cases of submandibular duct involvement (2). If the clinical examination indicates suspicion of obstructive salivary gland pathology, panoramic and occlusal X-rays are ordered for submandibular gland assessment (6). Patients suffering from aggressive sialadenitis, possibly resulting from obstruction of gland, are hospitalized and treated with intravenous antibiotics. During hospitalization, a CT scan is usually ordered. After the acute phase, sialography of the affected gland is routinely carried out (6).

There are numerous imaging techniques for salivary glands such as ultrasonography (US), magnetic resonance imaging (MRI), contrast enhanced computed tomography (CECT), conventional and subtraction sialography, salivary gland scintigraphy and magnetic resonance sialography (MRS). Improvement in imaging techniques (US, CT and MRI) has reduced indications for conventional sialography. However, sialectasis, radiopaque sialoliths and post inflammatory ductal strictures continue to be the best visualized with digital subtraction sialography. Furthermore, US, CT and MRI have the advantage of depicting the glandular parenchyma in addition to depicting sialolithiasis and changes of ductal structure (7). Sialography is a quick, widely available and excellent study of extra-glandular and intra-glandular ducts of parotid and submandibular glands by retrograde injection of iodinated contrast (8). Sialography is considered a simple procedure and if carried out properly, no local anesthesia is required in most cases. The procedure is carried out on all patients in an ambulatory setting (6). Hypersensitivity to the contrast agent, acute infections, thyroid and uncooperative patient are contraindications for sialography (8).

To date, there has been no reported case of salivary duct perforation due to sialography as evident by extensive MEDLINE, Pre-MEDLINE and English literature search. In this case, 3.0 mL of contrast agent was injected while the amount of contrast agent required for submandibular sialography is only about 0.5 to 1.2 mL. Thus, over infusion with subsequent over distension of the ductal system was postulated as the cause of rupture, although the operator did not feel any resistance and the patient tolerated the procedure well. We suggest that more attention should be paid during sialography to prevent similar incidences and the operators should be informed about how much contrast agent should be infused as depicted in published data. Fortunately, most extravasations result in minimal swelling or erythema, with no long-term sequelae; however, severe skin necrosis and ulceration may occur (9). Conservative management with broad-spectrum antibiotics, short course of corticosteroids and analgesics are necessities for first-line management, which should be followed by definitive management of the respective pathological lesion once the acute problem is resolved.
